# The association between failed quit attempts and increased levels of psychological distress in smokers in a large New Zealand cohort

**DOI:** 10.1186/1471-2458-11-598

**Published:** 2011-07-28

**Authors:** Frederieke S van der Deen, Kristie N Carter, Nick Wilson, Sunny Collings

**Affiliations:** 1 Health Inequalities Research Programme, Department of Public Health, University of Otago, Wellington, P.O. Box 7343, Wellington South 6242, New Zealand; 2 Department of Health & Society, Leeuwenborch, Wageningen University, P.O. Box 9101, Hollandseweg 1, 6706 KN Wageningen, The Netherlands; 3 Department of Public Health, University of Otago, Wellington, P.O. Box 7343, Wellington South 6242, New Zealand; 4 Social Psychiatry & Population Mental Health Unit, University of Otago, Wellington, P.O. Box 7343, Wellington South 6242, New Zealand

**Keywords:** smoking status, quit status, psychological distress, socioeconomic status

## Abstract

**Background:**

Although the association between smoking status and poorer mental health has been well documented, the association between quit status and psychological distress is less clear. The aim of the present study is to investigate the association of smoking status and quit status with psychological distress.

**Methods:**

Data for this study is from a single year of the Survey of Families, Income and Employment (SoFIE) conducted in New Zealand (2004/05) (n = 18,525 respondents). Smoking status and quit status were treated as exposure variables, and psychological distress (Kessler-10) was treated as the outcome variable. Logistic regression analyses were performed to determine the association of smoking with psychological distress in the whole adult population and quit status with psychological distress in the ex- and current-smoking population.

**Results:**

Current smokers had higher rates of high and very high psychological distress compared to never smokers (adjusted odds ratio (aOR) = 1.45; 95% CI: 1.24-1.69). Unsuccessful quitters had much higher levels of high to very high levels of psychological distress (16%) than any other group. Moreover, compared to long-term ex-smokers, unsuccessful quitters had a much higher odds of high to very high levels of psychological distress (aOR = 1.73; 95% CI: 1.36-2.21).

**Conclusion:**

These findings suggest that the significant association between smoking and psychological distress might be partly explained by increased levels of psychological distress among current smokers who made a quit attempt in the last year. This issue needs further study as it has implications for optimising the design of quitting support.

## Background

A number of national health surveys have shown that people with poorer mental health are more likely to be current smokers, be more heavily dependent on nicotine, have greater difficulty to successfully quit smoking and consequently be more likely to develop smoking-related illnesses than people with better mental health status [1-5].

The existing literature on the associations between depression and smoking quit rates is conflicting. Although some studies have not shown associations between history of depression and quit rates or the motivation and intent to quit [6-8], it has frequently been shown that people with depressive symptoms are less likely to successfully abstain from smoking [9-11]. Also, smokers with a history of major depression who have successfully quit have a higher risk of developing new depressive symptoms for a period of at least six months post-quitting [12]. However, another study found that successful quitting was not associated with an increased incidence of major depressive episodes, while not staying successfully abstinent after a quit attempt was [13]. A recent study following a cohort of smokers showed that longer-term smoking abstinence (9 months) did not lead to an increased risk of depressive symptoms or anxiety [14]. However, this study had a small number of quitters and did not examine those who had attempted but failed to quit smoking.

Recently, it was found in the US that current smokers who attempt but fail to quit report higher mean levels of serious psychological distress than others (i.e., those who have never smoked, or who are former smokers or current smokers who have not tried to quit) [15]. Current smoking and unsuccessful quit attempts in the last 12 months were in another study also associated with high levels of psychological distress [16]. In addition, this study showed that the longer a person had successfully quit smoking, the more similar their levels of psychological distress were compared to a never smoker [16]. Another study in which the relationship between smoking cessation and depression was investigated showed that the prevalence of lifetime depression was highest among unsuccessful quitters compared to those smokers who had not tried to quit and ex-smokers [17]. The fact that long-term ex-smokers are less anxious and depressed than current smokers may be because people who successfully quit smoking have better mental health overall [18].

In New Zealand (NZ), it has been estimated that approximately 33% of smoking occurs among those with mental disorders [19]. Moreover, the prevalence of such disorders among adult smokers is higher than in adult non-smokers and compared to the general population [20]. Currently, tobacco cigarette smoking is one of the leading causes of preventable mortality in NZ as in many other developed countries [21,22]. In addition, the lifetime prevalence of mental disorders is moderately high in NZ-almost 50% of the population will meet criteria for an anxiety, mood, substance use or eating disorder at one point over the life course [23].

A better understanding of the nature of the association between mental health and both smoking and quitting could enable the development of better targeted and more appropriate smoking cessation interventions. The objectives of the present study are therefore twofold. The first is to examine the association between smoking status and psychological distress using data from a NZ nationally representative survey. The second is to examine the association between the history of quitting behaviours and psychological distress in ex- and current- smokers.

## Methods

### Data

This study is a cross-sectional analysis of Wave 3 (2004) data from the Survey of Families, Income and Employment (SoFIE) which was conducted from 2002 to 2010 (SoFIE data Wave 1 to 7 version 1). In short, SoFIE is a nationally representative longitudinal survey of the usually resident population living in private households in NZ [24]. During annual face-to-face interviews, information was obtained on individual and family factors such as labour market activity, education, marital status and household income. In Wave 3 of the survey, a detailed health module included questions on health-related quality of life, mental health, chronic disease and health behaviours. The initial sample comprised approximately 11,500 responding private households (response rate of 77%) with 22,270 adults responding in Wave 1 which reduced to 20,375 in Wave 2 and 19,225 in Wave 3. The final analysis sample was restricted to 18,525 adult respondents at Wave 3, as there were 700 people with missing smoking information.

### Measures

#### Exposures

##### Smoking status

For the first objective of the study, the exposure measure was smoking status classified as "never", "ex" or "current" smoker. Responses to two questions about tobacco cigarette smoking were used to determine the respondents' smoking status. The first question was: "*Do you regularly smoke one or more tobacco cigarettes a day?*" A person was classified as "never smoker" (reference group) if he/she had never regularly smoked one or more cigarettes a day; as "ex-smoker" if he/she reported not regularly smoking but having *"ever been a regular smoker of one or more cigarettes a day"; *and finally as "current smoker" if he/she reported currently smoking one or more cigarettes a day on a regular basis. "Tobacco cigarette" was self-defined (there was no differentiation between store-bought and roll-your-own cigarettes or type of cigarette smoked).

##### Quit status

For the second objective, the exposure of interest was quit status, using only current and ex-smokers for this analysis. The quit status of the respondents was assessed by asking current smokers: "*In the last 12 months, have you tried to stop smoking altogether*?" and ex-smokers: "*How old were you the last time you quit smoking*?" Using the information on temporality of quit behaviours, we created a four-level quit status variable, defined as: (i) "long-term ex-smokers" (reference group) including ex-smokers who successfully quit smoking five or more years ago; (ii) "recent ex-smokers" including ex-smokers who quit within the last five years; (iii) "unsuccesful quitters" including current smokers with a failed quit attempt in the 12 months preceding the interview; and (iv) "non-quitters" including current smokers who had made no attempt to quit in the previous 12 months.

#### Outcome

##### Psychological distress

For all of the analyses the main outcome of interest was the level of non-specific psychological distress at Wave 3. This was measured by the Kessler Psychological Distress Scale (Kessler-10) [25-27], a 10-item questionnaire with questions regarding negative emotional states (e.g., feeling nervous, hopeless or worthless) in the previous four weeks. For analysis the Kessler-10 scores were grouped into four levels: "low" (score of 10-15), "moderate" (score of 16-21), "high" (score of 22-29) and "very high" (score of 30 or higher) [25,28]. For regression analyses these were dichotomised into low to moderate versus high to very high levels of psychological distress.

#### Other variables

Data on demographic confounders were taken from the Wave 3 interview: age, sex, prioritised ethnicity (NZ/European, Māori, Pacific, Asian, other) [29] and legal marital status (never married, divorced/separated/widowed, and legally married).

Socioeconomic factors considered to be confounders were: labour market activity (employed, not employed but seeking work, or not employed and not seeking work); highest educational qualification (no school qualification, school qualification, post-school vocational qualification, degree/higher qualification); equivalised household income (categorised into quintiles); and two measures of deprivation. The first was the small area specific deprivation index (NZDep) [30]. The second measure of deprivation was the NZ-specific individual deprivation index (NZiDep), a measure of the individual socioeconomic position determined from eight "yes or no" questions about financial deprivation (e.g. use of food grants or the need to borrow money for day-to-day needs) [31]. The scores for NZiDep are grouped into five values, ranging from one (no deprivation factors) to five (more than 5 deprivation factors) [31]. We chose to adjust for these socioeconomic confounders in the present analyses as people with lower socioeconomic status are known to have an increased risk of ever taking up smoking and less likely to give up on smoking [32-34]. Moreover, in NZ it has been shown that prevalence of mental disorders is higher among those with lower socioeconomic status (as measured by education, household income and small area index of deprivation) [23].

### Statistical analysis

All analyses were conducted using individual unit data in SAS 8.2 in the Statistics NZ data laboratory. Logistic regression was used to explore the association of smoking status (objective 1) and quit status (objective 2) with high to very high levels of psychological distress compared to low to moderate levels of psychological distress. Models were built sequentially by adjusting first for demographic confounders, then adjusting for socioeconomic confounders and finally adjusting for NZiDep in the model.

## Results

### Smoking status and psychological distress

Of the 18,525 respondents, 20% were current smokers, 25% were ex-smokers and 55% were never smokers (Table 1). Compared to never smokers, current smokers were more likely to be male, of younger age, Māori ethnicity, never married, have lower educational qualifications, lower incomes, live in deprived neighbourhoods, and report more factors of individual deprivation. Current smokers were more likely to report high to very high levels of psychological distress than ex- or never-smokers. Logistic regression models (Table 2) show that compared to never smokers, the adjusted odds ratio (aOR) of reporting high to very high levels of psychological distress was significantly greater for current smokers (model 4: OR: 1.45; 95% CI: 1.24-1.69); and marginally significant for ex-smokers (aOR: 1.18; 95% CI: 1.00-1.39).

**Table 1:** Demographic and socioeconomic variables by smoking and quit status

	All	Never smoker			Ex-smoker quit > = 5 yrs			Ex-smoker quit < 5 yrs			Current smoker tried to quit			Current smoker not tried to quit		
	N	N	row%	col%	N	row%	col%	N	row%	col%	N	row%	col%	N	row%	col%
**All**	18525	10110	54.6		3595	19.4		1065	5.8		1470	7.9		2285	12.3	
**Kessler 10**																
Low + moderate	17120	9495	55.5	93.9	3360	19.6	93.5	955	5.6	89.7	1225	7.2	83.3	2055	12.0	89.9
High + very high	1270	530	41.7	5.2	200	15.8	5.6	100	7.9	9.4	230	18.1	15.7	205	16.1	9.0
**Sex**																
Female	10340	5680	54.9	56.2	1725	16.7	48.0	585	5.7	54.9	835	8.1	56.8	1160	11.2	50.8
Male	8885	4430	49.9	43.8	1870	21.1	52.0	480	5.4	45.1	635	7.2	43.2	1125	12.7	49.2
**Age group (years)**																
15-24	3135	2160	68.9	21.4	25	0.8	0.7	165	5.3	15.5	285	9.1	19.4	345	11.0	15.1
25-44	6515	3350	51.4	33.1	775	11.9	21.6	510	7.8	47.9	650	10.0	44.2	980	15.0	42.9
45-64	6345	3025	47.7	29.9	1575	24.8	43.8	290	4.6	27.2	450	7.1	30.6	795	12.5	34.8
65+	3230	1575	48.8	15.6	1220	37.8	33.9	95	2.9	8.9	85	2.6	5.8	165	5.1	7.2
**Ethnicity**																
NZ/European	14495	7705	53.2	76.2	3035	20.9	84.4	810	5.6	76.1	940	6.5	64.0	1580	10.9	69.2
Māori	2375	860	36.2	8.5	350	14.7	9.7	155	6.5	14.6	360	15.2	24.5	520	21.9	22.8
Pacific	900	515	57.2	5.1	75	8.3	2.1	45	5.0	4.2	95	10.6	6.5	95	10.6	4.2
Asian	1010	775	76.7	7.7	55	5.5	1.5	30	3.0	2.8	50	5.0	3.4	50	5.0	2.2
Other	440	255	58.0	2.5	75	17.1	2.1	25	5.7	2.4	30	6.8	2.0	35	8.0	1.5
**Marital status**																
Married	9610	5260	54.7	52.0	2415	25.1	67.2	490	5.1	46.0	510	5.3	34.7	890	9.3	39.0
Divorced, widowed, separated	3235	1430	44.2	14.1	825	25.5	23.0	180	5.6	16.9	330	10.2	22.5	455	14.1	19.9
Never married	5770	3415	59.2	33.8	355	6.2	9.9	395	6.9	37.1	635	11.0	43.2	935	16.2	40.9
**Maximum educational level**																
Degree	2705	1835	67.8	18.2	425	15.7	11.8	120	4.4	11.3	90	3.3	6.1	135	5.0	5.9
Post-school qualification	6495	3165	48.7	31.3	1375	21.2	38.3	385	5.9	36.2	540	8.3	36.7	825	12.7	36.1
School qualification	5165	2950	57.1	29.2	760	14.7	21.1	325	6.3	30.5	375	7.3	25.5	575	11.1	25.2
No qualification	4855	2160	44.5	21.4	1030	21.2	28.7	235	4.8	22.1	465	9.6	31.6	745	15.4	32.6
**Labour market activity**																
Working	12435	6610	53.2	65.4	2075	16.7	57.7	750	6.0	70.4	965	7.8	65.7	1605	12.9	70.2
Not employed, looking for work	370	150	40.5	1.5	35	9.5	1.0	20	5.4	1.9	55	14.9	3.7	90	24.3	3.9
Not employed, not looking for work	6395	3350	52.4	33.1	1485	23.2	41.3	295	4.6	27.7	450	7.0	30.6	590	9.2	25.8
**NZDep2001 area unit (quintiles)**																
NZDepQ1 (least deprived)	3860	2455	63.6	24.3	770	20.0	21.4	185	4.8	17.4	155	4.0	10.5	240	6.2	10.5
NZDepQ2	3830	2220	58.0	22.0	790	20.6	22.0	205	5.4	19.3	215	5.6	14.6	335	8.8	14.7
NZDepQ3	3440	1840	53.5	18.2	635	18.5	17.7	210	6.1	19.7	285	8.3	19.4	430	12.5	18.8
NZDepQ4	4000	2000	50.0	19.8	785	19.6	21.8	245	6.1	23.0	340	8.5	23.1	565	14.1	24.7
NZDepQ5 (most deprived)	3690	1595	43.2	15.8	610	16.5	17.0	220	6.0	20.7	475	12.9	32.3	715	19.4	31.3
**Household income (quintiles)**																
Q1 (lowest income)	3845	1620	42.1	16.0	585	15.2	16.3	195	5.1	18.3	415	10.8	28.2	505	13.1	22.1
Q2	3845	1910	49.7	18.9	880	22.9	24.5	210	5.5	19.7	335	8.7	22.8	470	12.2	20.6
Q3	3845	2075	54.0	20.5	695	18.1	19.3	210	5.5	19.7	290	7.5	19.7	525	13.7	23.0
Q4	3845	2160	56.2	21.4	710	18.5	19.8	230	6.0	21.6	240	6.2	16.3	470	12.2	20.6
Q5 (highest income)	3845	2345	61.0	23.2	725	18.9	20.2	220	5.7	20.7	190	5.0	12.9	310	8.1	13.6
**NZiDep**																
0 individual deprivation factors	13370	7810	58.4	77.3	2795	20.9	77.8	675	5.1	63.4	720	5.4	49.0	1355	10.1	59.3
1 individual deprivation factors	2790	1400	50.2	13.9	460	16.5	12.8	195	7.0	18.3	305	10.9	20.8	425	15.2	18.6
2 individual deprivation factors	1110	480	43.2	4.8	170	15.3	4.7	90	8.1	8.5	145	13.1	9.9	225	20.3	9.9
3-4 individual deprivation factors	980	350	35.7	3.5	140	14.3	3.9	75	7.7	7.0	205	20.9	14.0	210	21.4	9.2
5+ individual deprivation factors	295	60	20.3	0.6	35	11.9	1.0	25	8.5	2.4	100	33.9	6.8	75	25.4	3.3

**Table 2:** Logistic regression odds ratios (OR) and 95% confidence intervals of high to very high levels of psychological distress by smoking status

Smoking status	Model 1		Model 2		Model 3		Model 4	
	OR	95% CI	aOR	95% CI	aOR	95% CI	aOR	95% CI
Never smokers (ref)	1		1		1		1	
Current smokers	2.44	(2.13-2.79)	2.21	(1.91-2.55)	1.82	(1.57-2.11)	1.45	(1.24-1.69)
Ex-smokers	1.28	(1.10-1.48)	1.42	(1.21-1.66)	1.33	(1.13-1.56)	1.18	(1.00-1.39)

Model 1 = Crude model

Model 2 = Adjusted for Age, Sex, Prioritised Ethnicity and Marital Status

Model 3 = Adjusted as per Model 2 plus: Household Income, Labour Market Activity, Educational Qualification and NZ (area) Deprivation

Model 4 = Adjusted as per Model 3 plus: NZ individual deprivation (NZiDep)

### Quit status and psychological distress

Of the 8,415 current and ex-smoker respondents included in the Objective 2 analysis, 43% were long-term ex-smokers (77% of ex-smokers), 13% were recent ex-smokers (23% of ex-smokers), 17% were unsuccessful quitters (39% of current smokers) and 27% were non-quitters (61% of current smokers) (Table 1). Unsuccessful quitters were more likely to be female, had lower incomes and reported more individual deprivation factors. Moreover, unsuccessful quitters had much higher levels of high or very high levels of psychological distress (16%) than any other group.

In the logistic regression analyses (Table 3), recent ex-smokers (who had quit in the past five years) had elevated odds of high to very high levels of psychological distress in all four models compared to long-term ex-smokers (model 4: aOR = 1.33, 95% CI: 1.00-1.76). This pattern was similar for non-quitters (compared to long-term ex-smokers). The odds of high to very high levels of psychological distress were significantly greater for unsuccessful quitters compared to long-term ex-smokers (model 4: aOR = 1.73, 95% CI: 1.36-2.21), although the 95% confidence interval overlapped with that for recent ex-smokers (and non-quitters).

**Table 3:** Logistic regression odds ratios (OR) and 95% confidence intervals of high to very high levels of psychological distress by quit status (in ex and current smokers only)

Smoking and quit status	Model 1		Model 2		Model 3		Model 4	
	OR	95% CI	aOR	95% CI	aOR	95% CI	aOR	95% CI
Ex-smoker, quit > = 5 years ago (reference)	1		1		1		1	
Ex-smoker, quit < 5 years ago	1.67	(1.30-2.16)	1.42	(1.08-1.86)	1.39	(1.05-1.82)	1.33	(1.00-1.76)
Current smoker tried to quit (last 12 months)	3.06	(2.50-3.75	2.37	(1.89-2.97)	2.02	(1.60-2.55)	1.73	(1.36-2.21)
Current smoker, not tried to quit (last 12 months)	1.65	(1.35-2.02)	1.34	(1.07-1.68)	1.20	(0.95-1.51)	1.16	(0.92-1.47)

Model 1 = Crude model

Model 2 = Adjusted for Age, Sex, Prioritised Ethnicity and Marital Status

Model 3 = Adjusted as per Model 2 plus: Household Income, Labour Market Activity, Educational Qualification and NZ (area) Deprivation

Model 4 = Adjusted as per Model 3 plus: NZ individual deprivation (NZiDep)

## Discussion

After adjusting for a wide range of demographic and socioeconomic variables, we found a strong relationship between smoking status and psychological distress, with current smokers being almost 50% more likely to report higher levels of psychological distress than never smokers. The uniqueness of the SoFIE data allowed us to investigate in more detail the association between quitting smoking and psychological distress while adjusting for a range of demographic and socioeconomic variables. Compared to long-term ex-smokers (who had quit 5 or more years ago) we also found elevated odds for high to very high levels of psychological distress in recent ex-smokers, and non-quitters (significant in most but not all models). However, the odds appeared to be raised more in unsuccessful quitters, although the confidence intervals overlapped. These findings have a number of methodological and public health implications.

### Methodological implications

The observed association between smoking status and psychological distress among current smokers may be largely explained by the increased levels of psychological distress in current smokers who recently made an attempt to quit smoking but failed. It is well known that smoking cessation can be a difficult process with several quit attempts often preceding long-term abstinence [35]. While longer-term abstinence may not be associated with an increase in depressive or anxiety symptoms [14], our study shows that unsuccessful quitting is strongly associated with high levels of psychological distress. Therefore, failing to take recent unsuccessful quit attempts into account in analyses of smoking and mental health status may lead to misleading results. A recent longitudinal study from NZ argued that cigarette smoking increases the risk of depressive symptoms [36]. However, this study did not take into account recent quit attempts. Our results suggest that attempts at quitting smoking may partly explain the relationship between smoking and symptoms of mental disorders. However, this relationship is complex and modelling it is challenging.

### Public health implications

Although the apparent occurrence of increased levels of psychological distress in unsuccessful quitters needs further study, we suggest in the meantime that health professionals take a precautionary approach and assume that this elevated distress could potentially be ameliorated by raising the success rate of quitting amongst these smokers. This could be achieved by strengthening population-level tobacco control measures that support quitting and staying quit (e.g., high tobacco taxes and restrictions on tobacco marketing). It would also be assisted by the tailoring of quitting support for those with elevated psychological distress. To assess psychological distress among clients, quitting support (face-to-face, quit lines and web-based services) could use the Kessler-10 scale and adapt their services to the outcome of this measure. In addition, the value of successful quitting as a means to lower psychological distress can potentially be highlighted.

### Strengths and limitations of this study

The main strengths of this study were the large sample size and the ability to adjust for a wide range of potential demographic and socioeconomic confounders in regression modelling. We used the Kessler-10 measure of psychological distress, which is used as a screening tool for mental health morbidity at the population level [37]. It gives a measure of experienced non-specific psychological distress on a continuum rather than a clinical disease classification and findings of the present study are therefore of greater relevance in informing national population-level services and national public health messages. It has been shown that high and very high scores on the Kessler-10 are associated with clinical measures of anxiety and depressive disorders [25,38]. The previous US study that found a strong relationship between unsuccessful quit attempts and high levels of psychological distress, used the Kessler-6 scale [15] which is not as robust as the Kessler-10. Also, in this study the authors could not establish a relationship between the history of quit behaviours and psychological distress, due to a lack of information on how long ago respondents had quit smoking or when they had made a quit attempt [15]. Therefore, one of the strengths of our study is that the SoFIE dataset contains information on how long ago ex-smokers quit smoking, and therefore we were able to construct a variable for quitting smoking over different time intervals.

Our findings are supported by a recent study in which the association between smoking, quitting and psychological distress was investigated [16]. Although the authors of this study were also able to construct an interval variable for quit status and found a much stronger relationship between failed quit attempts and psychological distress, the uniqueness of the SoFIE data enabled us to adjust this relationship for a wider range of demographic and socioeconomic variables (e.g., prioritised ethnicity, marital status, an individual measure of deprivation (NZiDep), labour market activity and household income). In addition we consider that having a referent group of long term ex-smokers (who quit five or more years ago) is potentially more appropriate than using never smokers.

Several limitations need to be considered in the interpretation of our results. Firstly, this was a cross-sectional (Wave 3) analysis, using data from a longitudinal survey. Therefore we cannot infer causality in the direction of the relationship i.e. if the failure of quitting smoking causes higher levels of psychological distress or that higher levels of psychological distress cause failure in quitting smoking. Future research using the longitudinal SoFIE data will allow more in-depth analyses of the causal pathway between quitting behaviour and psychological distress. Although the original SoFIE study was a nationally representative survey the first module health data was collected in Wave 3 with a response rate of 77% of the original adult population (at Wave 1). It has been shown that younger people of lower socioeconomic status are more likely to drop out of the survey [24]. This sample attrition may have led to selection bias and negatively influenced the generalisability of the study results. However, unless the dropout rates were jointly distributed by smoking or quit status and psychological distress, we argue that the effect of attrition on our exposure-outcome relationship would have been minimal.

There are also some limitations to the smoking questions in the survey. There was no differentiation between types of cigarettes or tobacco consumed. However, recently a NZ study showed that quitting behaviour did not differ by the type of consumed cigarettes [39]. Also no information was collected on quit attempts more than 12 months ago, or specific quit strategies in unsuccessful attempts. Moreover, questions on smoking can potentially be sensitive, and so there may be some information (social desirability) bias present (especially given evidence around smoking denormalisation in NZ in recent years [40]. However, our proportion of current smokers (20%) is the same as that shown in the NZ Health Survey 2006/2007 and 2006 Census and recently it has been shown that self-reports of smoking during face-to-face surveys are quite accurate [41-43]. Finally, there might be unmeasured confounders influencing the relationship between smoking, attempting to quit smoking and mental health such as family support, the household environment or previous quit attempts. Not controlling for these factors may have led to an overestimate of the true association.

## Conclusion

Our findings suggest that the significant association between smoking and psychological distress might be partly due to increased levels of psychological distress in current smokers who tried to quit in the last year. This issue needs further study as it may have important implications for optimising the design of quitting support provided by family members and health services.

## Competing interests

The authors declare that they have no competing interests.

## Authors' contributions

FSvdD performed literature study, participated in statistical analysis, and drafted manuscript. KNC designed study, performed statistical analysis, and helped drafting manuscript. NW was involved in design study and participated in interpreting results. SC participated in interpreting results. All authors read and approved the manuscript.

## Pre-publication history

The pre-publication history for this paper can be accessed here:

http://www.biomedcentral.com/1471-2458/11/598/prepub
